# Anxiolytic-like effects of acute serotonin-releasing agents in zebrafish models of anxiety: experimental study and systematic review

**DOI:** 10.1017/neu.2024.44

**Published:** 2024-10-28

**Authors:** Jakob Näslund, Jenny Landin, Fredrik Hieronymus, Rakesh Kumar Banote, Petronella Kettunen

**Affiliations:** 1 Department of Pharmacology, Institute of Neuroscience and Physiology at the Sahlgrenska Academy, University of Gothenburg, Gothenburg, Sweden; 2 Department of Psychiatry and Neurochemistry, Institute of Neuroscience and Physiology at the Sahlgrenska Academy, University of Gothenburg, Gothenburg, Sweden; 3 Department of Neurology, Sahlgrenska University Hosp1ital, Gothenburg, Sweden; 4 Department of Clinical Neuroscience, Sahlgrenska Academy, University of Gothenburg, Sweden; 5 Nuffield Department of Clinical Neurosciences, John Radcliffe Hospital, University of Oxford, Oxford, UK

**Keywords:** Serotonin, SSRI, anxiety, zebrafish

## Abstract

Though commonly used to model affective disorders, zebrafish display notable differences in terms of the structure and function of the brain serotonin system, including responses to pharmacological interventions, as compared to mammals. For example, elevation of brain serotonin following acute administration of serotonin reuptake inhibitors (SRIs) generally has anxiogenic effects, both in the clinical situation and in rodent models of anxiety, but previous research has indicated the opposite in zebrafish. However, several issues remain unresolved. We conducted a systematic review of SRI effects in zebrafish models of anxiety and, on the basis of these results, performed a series of experiments further investigating the influence of serotonin-releasing agents on anxiety-like behaviour in zebrafish, with sex-segregated wild-type animals being administered either escitalopram, or the serotonin releaser fenfluramine, in the light-dark test. In the systematic review, we find that the available literature indicates an anxiolytic-like effect of SRIs in the novel-tank diving test. Regarding the light-dark test, most studies reported no behavioural effects of SRIs, although the few that did generally saw anxiolytic-like responses. In the experimental studies, consistent anxiolytic-like effects were observed with neither sex nor habituation influencing treatment response. We find that the general effect of acute SRI administration in zebrafish indeed appears to be anxiolytic-like, indicating, at least partly, differences in the functioning of the serotonin system as compared to mammals and that caution is advised when using zebrafish to model affective disorders.

Significant outcomes

- Published zebrafish studies employing the novel-tank diving test almost unequivocally indicate an anxiolytic-like effect of acute serotonin elevation, in contrast with rodent studies and the clinical situation. For the other major anxiety test in zebrafish, the light-dark test, the literature is less comprehensive, and the effects of acute SRI treatment are less clear, possibly due to sub-optimal doses often being used.
- Results from the experimental study here reported that investigated a selective serotonin reuptake inhibitor (SSRI), as well as the serotonin releaser fenfluramine, in the light-dark test while controlling for possible effects of sex and habituation indicate that the effects of acute serotonin elevation also in this test are anxiolytic-like. This (as well as several lines of evidence from earlier work) indicates significant differences in terms of the behavioural pharmacology of the serotonin system in zebrafish as compared to mammals.

Limitations

- For 3R reasons, animals were re-used; however, a separate experiment conducted beforehand indicated that this was unlikely to impact SSRI responses in the light-dark test, especially considering the amount of time allowed to elapse between each session.
- Though individual differences in temperament may influence responses to pharmacological agents, we neither assayed baseline temperament differences nor tagged animals in order to track the performance of individual fish across the experiments.

## Highlights

- Zebrafish appear to behave differently to mammals when given drugs that rapidly increase serotonin levels: while rodents and humans react with higher levels of fear or anxiety, zebrafish instead appear *less* ‘anxious’.
- This seems to hold also when controlling for sex, previous experience of the testing situation and across different classes of drugs.
- This may indicate that zebrafish are a less-than-optimal choice of model organism when studying, for example, anxiety and depression, especially considering other differences in terms of the functioning of the serotonin system between zebrafish and mammals.

## Introduction

Despite decades of research, assessing the validity of animal models of psychiatric disorders remains a difficult task. This reflects both a limited understanding of the pathophysiology of these conditions and a paucity of reliable biological correlates for observed symptoms. For affective disorders in particular, there exist only a few biological correlates with (limited) research utility, such as aberrant REM sleep patterns and decreased autonomic variability in patients with depression (Stein *et al*., 2000; Palagini *et al*., 2013) and reduced suppression of cortisol secretion by dexamethasone in those with melancholic depression (Carroll, 1981). Moreover, the presence of such markers is, in general, neither pathognomonic nor common to more than a subset of patients (Nierenberg & Feinstein, 1988; Arfken *et al*., 2014; García-Gutiérrez *et al*., 2020). Behavioural responses to pharmacological interventions thus remain the primary means to assess the validity of putative animal models of affective disorders. Ideally then, substances that are anxiolytic in man should register as anxiolytic-like in an animal model, antidepressants should register as antidepressant-like and so on. For example, while long-term administration of a serotonin reuptake inhibitor (SRI) is an effective treatment for anxiety disorders (Bandelow & Michaelis, 2015), they do not provide immediate relief in the manner of benzodiazepines – if anything, they *increase* anxiety after the first dose(s) (Sinclair *et al*., 2009; Näslund *et al*., 2017), with a therapeutic effect beginning to emerge after roughly a week of treatment (Hieronymus *et al*., 2016). This difference between the acute and chronic effects of SRIs is largely mirrored by rodent models of anxiety-like behaviour (see, e.g. Mombereu and co-workers for a more thorough discussion [Mombereau *et al*., 2010]).

The zebrafish has seen increased use as a model organism in pre-clinical psychiatric research, but some outstanding questions remain regarding how well findings in zebrafish translate to mammalian models and to the clinical situation and vice versa. The overarching goal of the studies reported in this paper has been to investigate one such issue where discrepancies seem to be present, namely that of behavioural responses to acute administration of serotonin-elevating agents. Studies employing two of the major models of anxiety-like behaviour in zebrafish – the novel-tank diving (geotaxis) test and the light-dark (scototaxis) test – have reported somewhat conflicting results, especially for the latter, with both increased (Magno *et al*., 2015) and decreased (Benneh *et al*., 2017; Giacomini *et al*., 2016, 2020) anxiety-like behaviour being observed after acute SRI administration. Since serotonin is an important mediator of sex differences in rodents as well as in humans (for further discussion and references on this subject, we refer to earlier work by us [Näslund *et al*., 2013]), it is conceivable that some of these discrepancies could be related to differential SRI responses in males and females as most zebrafish studies on the acute effects of various SRIs have used mixed-sex groups. Another factor that possibly could influence results is habituation to the apparatus; at least in rodent models, it is well-known that prior exposure to tests aiming to measure anxiety-like behaviour generally reduces such behaviour upon re-test (File, 1993).

This two-part study thus aims to systematise what is known about, as well as further investigate, the impact of acute serotonergic interventions in zebrafish tests of anxiety-like behaviour. The first part is a systematic review providing an up-to-date summary of results from previous studies using the novel-tank diving and light-dark tests. These findings are then analysed in relation to data obtained from the second part of the study; a series of experiments in which we investigate the effects of administration of serotonin-elevating agents on behaviour in the scototaxis test, while attempting to control for the influence of sex, mode of action and habituation to the apparatus.

A central focus of the research group is to actively work with 3R practices, and as we desired to be able to re-use the animals in the main experiments (Experiments III–V), we performed a pilot study (Experiment I) investigating whether earlier acute administration of an SRI influences anxiety-like behaviour of zebrafish in the scototaxis test, both in the absence and presence of an SRI. In Experiments I, III and IV, we employed a selective serotonin reuptake inhibitor (SSRI), escitalopram. This was chosen as it is the most selective of all the SRIs in clinical use (i.e. possessing the least affinity for targets other than the serotonin transporter) (Sánchez & Hyttel, 1999), while also having a half-life of little more than a day (Søgaard *et al*., 2005), which can be contrasted to the SSRI most commonly used in zebrafish studies, fluoxetine, where the active metabolite has a half-life of up to 2 weeks (Preskorn, 1997) (after repeated administration – but whether zebrafish metabolise as quickly as humans is unknown) – such a long half-life could conceivably have long-term effects that impact fish health and behaviour in an unforeseen manner. In Experiment IV, we attempted to replicate the findings from Experiment III while allowing for a possible effect of habituation to be observed. In Experiment V, the serotonin-releasing agent fenfluramine, anxiogenic in man and anxiogenic-like in rodents (File & Guardiola-Lemaitre, 1988; Targum & Marshall, 1989), was employed (a dose-finding study for fenfluramine, Experiment II, had also been performed). This was partly done to control for the possibility that the observed effects in Experiments III and IV had been related to drug-specific effects (i.e. escitalopram having effects on targets other than the serotonin transporter, such as the σ receptor (Albayrak & Hashimoto, 2017)) and partly to control for the possibility that a class-specific effect unique to zebrafish could explain the reported discrepancies in terms of behavioural effects of acute SSRI administration and anxiety-like behaviour as compared to humans and other mammals.

In short, our main aims were to (i) systematically summarise previous work regarding acute effects of serotonin-releasing agents in the most common zebrafish models of anxiety, (ii) investigate to what extent administration of serotonin-releasing agents exerts an anxiolytic-like or anxiogenic-like effect in the light-dark test, (iii) investigate if any observed effects are consistent across different classes or serotonin-elevating agents and (iv) investigate if sex, or the novelty of the situation, can influence a possible treatment response.

## Experimental procedures

### Literature search and systematic review process

**Figure 1.**
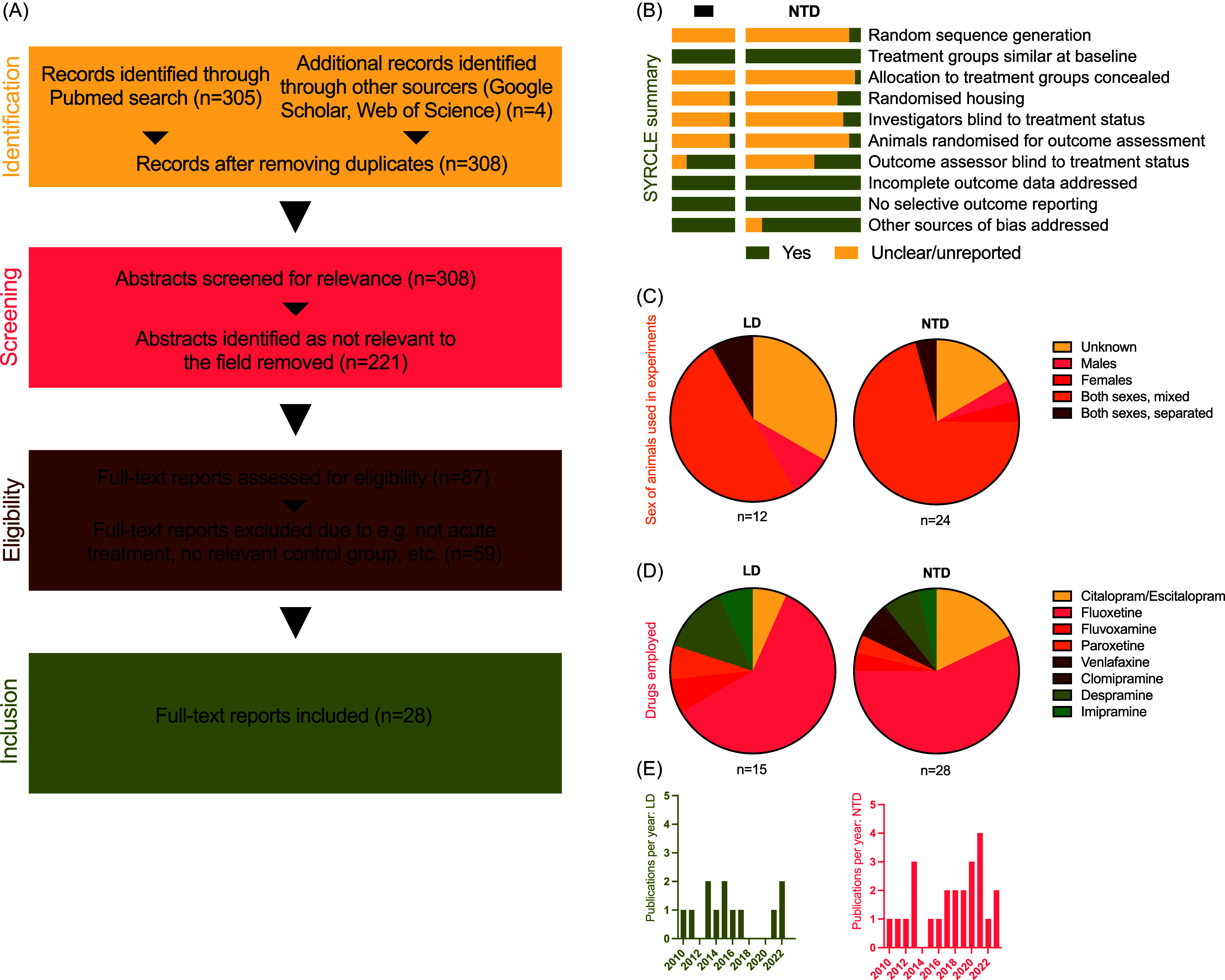
A ‘Preferred Reporting Items for Systematic reviews and Meta-analyses’ (PRISMA) flowchart illustrating the literature search process (A). Cumulative bar charts displaying risk of bias assessment across 10 items specified in SYRCLE’s assessment tool (B); due to the limited methodological descriptions of most articles, it was not possible to confidently classify any work as clearly *not* conforming to SYRCLE guidelines. Pie charts illustrating the distribution of relevant categories of sex (C) and substance used (D) in comparisons included in the systematic review. Histogram over number of identified studies published by year since 2010 (E). For items B–E, data are shown separately for the light-dark (LD, left) and novel-tank diving test (NTD, right).

A literature search was performed using PubMed/Medline, Web of Science and Google Scholar between December 01, 2021, and March 10, 2022, and repeated between June 1 and August 9, 2023 (Figure 1). Records published between 1945 and 2023 were included. For PubMed, the ‘Advanced search’ of titles and abstracts was used, for Web of Science, the ‘Abstract’ advanced search function was used and for Google Scholar, the ‘allintitle’ function. The search string was ([’zebrafish’or ‘danio rerio’ or ‘danio’] and [’citalopram’ or ‘escitalopram’ or ’sertraline’ or ‘zimelidine’ or ‘paroxetine’ or ‘fluoxetine’ or ‘fluvoxamine’ or ‘fenfluramine’ or ‘duloxetine’ or ‘venlafaxine’ or ‘fenfluramine’ or ’SRI’ or ’SSRI’ or ‘tricyclic’ or ‘amitriptyline’ or ‘imipramine’ or ‘clomipramine’ or ‘desipramine’]). Only studies employing adult zebrafish were considered. Regarding the inclusion of desipramine: note that it, in zebrafish, unlike in mammals, has a significant effect also on serotonin reuptake (Severinsen *et al*., 2008). Papers were assessed for eligibility by two independent reviewers, and subsequent quality assessment was performed using the ‘SYRCLE Risk of Bias Tool for Animal Studies’ (Hooijmans *et al*., 2014); here each paper is evaluated regarding risk of bias across 10 items, assigning a rating of high, low or unclear risk for each item. Any disagreement about a specific score was resolved through discussion and sometimes by consultation with a third reviewer.

### Animals

Male and female zebrafish of the wild-type AB line were used, having previously been procured from the Genome Editing Zebrafish facility, SciLifeLab, Uppsala University (Uppsala, Sweden) to the animal facility of the Department of Biological and Environmental Sciences at the University of Gothenburg. Animals in Experiment I were aged 12–18 months at the start of the experiment, while animals in Experiment II were aged 13–21 months. Animals used in Experiments III–V were aged 8–9 months at the start of Experiment III, before which males and females had been put into separate aquaria and left undisturbed for 4 weeks. Animals were housed in 10 l aquaria with continuous filtering, at a density of roughly 1 animal/l, under controlled conditions (conductivity 900 ± 10 µS, pH 7.4 ± 1, temperature of 27–28 °C) and a 14:10 h photoperiod with a gradual onset of lights at 07:45–08:00 a.m. and dimming of lights at 09:45–10:00 p.m.). Care was taken to ensure that all aquaria had similar surroundings and light levels and that they were equally likely to be disturbed during work in the housing room. Aquaria water was produced from Tropic Marine Pro-Reef Salt (Bioted Marine, Kungsbacka, Sweden) adjusted with bicarbonate. The aquaria water was changed twice per week and water quality was checked three times per week using test strips. The environment was enriched with one large and bushy artificial plant per aquarium, as well as images of stones below the aquaria. Fish were fed twice per day with a mixture of Tetra Pro Multi-Crisps flake food (Tetra Fish, Melle, Germany), granular pellets (ZM Fish foods, Winchester, UK) and hatched brine shrimp nauplii.

The authors assert that all procedures contributing to this work comply with the ethical standards of the relevant European, national and institutional guidelines on the care and use of laboratory animals. All experimental procedures and animal husbandry protocols also followed the Animal Research: Reporting of In Vivo Experiments (ARRIVE) guidelines and had been approved by the Gothenburg Animal Research Ethics Committee (case numbers 157-2015 and 5.8.18-08496/2018).

### Apparatus

Prior to testing in the light-dark apparatus and an hour before the start of any drug treatment, fish were moved from the housing room to the experiment room. Both rooms were lit by fluorescent lights, providing an illumination of roughly 1.5 mW/mm^2^ at the home aquaria, as well as at the bench where experiments were conducted. The light-dark test apparatus was similar to that of Maximino and co-workers (Maximino *et al*., 2010) and consisted of a half-black, half-white box (15 × 10 × 45 cm), with a 5 cm wide central compartment bound by sliding doors in which the animal was placed at test start. The apparatus was filled with fresh aquaria water, 10 cm deep. Fish were then incubated individually in 500 ml semi-translucent beakers spaced roughly 30 cm apart, filled with 250 ml fresh aquaria water or test solution (drug dissolved in fresh aquaria water) for 10 min. After drug incubation, the fish were netted into the central, closed holding chamber where they remained for 10 min before the sliding doors were removed, allowing the animal to explore the whole apparatus. They were then filmed for 15 min before being returned to the home aquaria. The apparatus was filled with fresh aquaria water of the same characteristics and temperature as described above, with water being changed after every animal. In order to assess if re-testing of animals in Experiments III–V would be likely to influence behaviour in subsequent tests, Experiment I was carried out to investigate whether acute treatment with an SSRI had any influence on anxiety-like behaviour when animals were re-exposed to the same drug 6 weeks later. No such effect was seen, but as an additional precaution, comparatively long intervals between Experiments III–V were chosen; 2 months separated Experiments III and IV, while Experiment V was conducted 4 months after Experiment IV. The shorter interval between Experiments III and IV was chosen to allow a possible habituation to the apparatus to be detected, but also this shorter interval was longer than that in Experiment I and is comparable to drug wash-out times in imaging studies in humans, where subjects often are their own controls In order to assess if re-testing of animals in Experiments III–V would be likely to influence behaviour in subsequent tests, Experiment I was carried out to investigate whether acute treatment with an SSRI had any influence on anxiety-like behaviour when animals were re-exposed to the same drug 6 weeks later. No such effect was seen, but as an additional precaution, comparatively long intervals between Experiments III–V were chosen; 2 months separated Experiments III and IV while Experiment V was conducted 4 months after Experiment IV. The shorter interval between Experiments III and IV was chosen to allow a possible habituation to the apparatus to be detected, but also this shorter interval was longer than that in Experiment I and is comparable to drug wash-out times in imaging studies in humans, where subjects often are their own controls (Klein *et al*., 2006; Lewis *et al*., 2021).

### Drugs

Escitalopram oxalate (Shodana Labs, Hyderabad, India) and *d*-fenfluramine hydrochloride (Tocris, Bristol, United Kingdom) were dissolved as a stock solution of fresh aquaria water before diluting them further in the working solutions. The escitalopram dose (50 mg/l, i.e. 154.09 µM) was selected from the published literature for the racemate, that is, citalopram (note that the R enantiomer of citalopram is practically inert; S-citalopram (i.e. escitalopram) is active) (Tables 1 and 2). We chose a slightly longer immersion time (10 min vs. 3–5 min in most published studies) as Sackerman and co-workers (Sackerman *et al*., 2010) reported comparatively low brain citalopram levels (about a third of the levels seen during treatment with standard doses in rodents) after 3 min of immersion in 100 mg/l of citalopram (i.e. equivalent to 50 mg/l of escitalopram). We chose to include a lower dose of escitalopram in Experiment III than that used in Experiment I as we in an unrelated experiment had found no differences between 25 and 50 mg/l in terms of anxiolytic-like effect and wanted to confirm this, as the possible use of a lower dose was considered desirable from an animal health perspective.

**Table 1.** Reported effects of acute SRI administration in the novel-tank diving, or geotaxis, test using adult zebrafish

Table 1: Novel-tank/geotaxis test		Time				Indices of anxiety-like behaviour		
Paper	Drugs (administration, doses)	Immersion (if applicable) +delay/habituation+test duration	Sex	Strain	N	Time in upper portion	Latency to first entry	Notes
Alves *et al*., 2023	Venlafaxine (37,5 mg)	30 min + 0 min + 6 min	M + F	WT (?)	14	↑	/	Commercial venlafaxine capsules dissolved in water
	Venlafaxine (75 mg)					↑	/	
	Venlafaxine (150 mg)					↑	/	
Benneh *et al*., 2017	Fluoxetine (0.03 mg/l)	20 min + 0? min + 5 min	?	WT (?)	?	→	→	Subsequent experiments had *n* = 5-8, but unclear what the group sizes were for this particular experiment
	Fluoxetine (0.1 mg/l)					↑	→	
	Fluoxetine (0.3 mg/l)					↑	↓	
	Desipramine (10 mg/l)					↑	↓	
	Desipramine (30 mg/l)					↑	↓	
	Desipramine (100 mg/l)					↑	↓	
Clément *et al*., 2020	Citalopram (30 mg/l)	5 min + 1 min + 10 min	M + F	?	?	↑	/	Conditioned place aversion (CPA) training employing a robotic heron for 4 days before treatment. Group sizes not stated, but with 64 subjects and equal groups n would be 8.
	Citalopram (50 mg/l)					↑	/	
	Citalopram (100 mg/l)					↑	/	
Costa de Melo *et al*., 2019	Fluoxetine (20 mg/l)	30 min + 60 min + 6 min	F	WT (AB)	4	↑/↑	/	Chronic unpredictable stress/social isolation
	Fluoxetine (p.o. 20 mg/kg)	N/A + 60 min + 6 min				↑/↑	/	
Demin *et al*., 2017	Amitriptyline (1 mg/l)	20 min + 0? min + 5 min	M + F	WT (shortfin)	15	↑	↓	
	Amitriptyline (5 mg/l)					↑	↓	
	Amitriptyline (10 mg/l)					→	→	
Fontana *et al*., 2022	Fluoxetine (100 μg/l)	30 min + 6 min + 5 min	M + F	WT (AB)	8	↑	/	
		30 min + 0 min + 6 min				↑	/	No habituation, but 6 min of NTD test before LDB
		30 min + 0 min + 6 min				↑	/	
Giacomini *et al*., 2016	Fluoxetine, 50 μg/l	15 min + 0? min + 6 min	M + F	WT (shortfin)	9–12	→	→	
Giacomini *et al*., 2020	Fluoxetine, 50 μg/l	60 min + 0 min + 6 min	M + F	WT (shortfin)	11–12	→	→	
Iturriaga-Vásquez *et al*., 2012	Fluoxetine (50 mg/l)	3 min + 5 min + 5min	?	?	?	↑	/	Very sparse information
	Fluoxetine (100 mg/l)					↑	/	
Karakaya *et al*., 2021	Citalopram (30 mg/l)	5 min + 1 min + 10 min	M + F	WT (?)	?	↑	/	CPA training employing a tight school of robotic zebrafish for 4 days before treatment. Group size unclear.
	Citalopram (100 mg/l)					↑	/	
Kulikova *et al*., 2021	Citalopram (0,25 mg/l)	180 min + 0? min + 5 min	M + F	WT (leopard)	18 (14)	→	/	14 in the control group, otherwise 18.
	Citalopram (0.5 mg/l)					→	/	
	Citalopram (0.1 mg/l)					→	/	
	Imipramine (0.25 mg/l)					→	/	
	Imipramine (0.5 mg/l)					↑	/	
	Imipramine (1 mg/l)					↑	/	
Lima-Maximino *et al*., 2020	Fluoxetine (i.p. 2.5 mg/kg)	N/A+(20 + 6 + 1) min + 6 min	M + F	WT (longfin)	14–15	↑	/	Complex pattern, but largely anxiolytic-like effects
	Fluoxetine (i.p. 25 mg/kg)					↑	/	
Macrì *et al*., 2019	Citalopram (30 mg/l)	5 min + 0 min + 6 min	M/F	WT (?)	16	↑	/	
	Citalopram (50 mg/l)					↑	/	
	Citalopram (100 mg/l)					↑	/	
Malyshev *et al*., 2021	Fluvoxamine (i.p. 5 mg/kg)	N/A + 10 min + 6 min	M + F	WT (shortfin)	18–30	↑	/	
Maximino *et al*., 2013a	Fluoxetine (i.p. 2.5 mg/kg)	N/A + 10 min + 6 min	?	WT (longfin)	10	↑	/	
	Fluoxetine (i.p. 5 mg/kg)					↑	/	
	Fluoxetine (i.p. 10 mg/kg)					↑	/	
Maximino *et al*., 2013b	Fluoxetine (i.p. 5 mg/kg)	N/A + (? min + 3 min) + 6 min	?	WT (longfin,)	9–10	↓	/	Both the light-dark test and novel-tank diving test were run; the order was random
	Fluoxetine (i.p 5 mg/kg)			WT (leopard)		↑	/	
Maximino *et al*., 2015	Fluoxetine (i.p. 1 mg/kg)	N/A + ? min + ? min	M	WT (striped longfin)	?	→	/	Size of the fluoxetine group unclear, but group sizes in other experiments reported in the paper are *n* = 7–12
Sabadin *et al*., 2022	Fluoxetine (100 (μg/l))	15 min + 0 min + 6 min	M + F	WT (?)	7	↑	/	Combined geo- and scototaxis test
Sackerman *et al*., 2010	Citalopram (100 mg/l)	3–4 min +? min + 5 min	M + F	ZIRC AB, GloFish, WIC, PETCO (WT/shortfin?)	9	↑	/	
	Desipramine (25 mg/l)				8	↑	/	
dos Santos Sampaio *et al*., 2018	Fluoxetine (p.o. 20 mg/kg)	N/A + 60 min + 6 min	M + F	WT (AB)	12	↑	↑	
Sinyakova *et al*., 2018	Fluoxetine (0.25 mg/l)	180 min + ? min + 5 min	M + F	NTL (?)	6 (12)	↑	/	12 in the control group, 6 in the SSRI group
Stewart *et al*., 2011	Fluoxetine (100 (μg/l))	20 min + ? min + 6 min?	M + F	WT (shortfin)	10–16	→	→	
	Fluoxetine (500 (μg/l))					→	→	
Stewart *et al*., 2013	Fluoxetine (1000 (μg/l))	60 min + ? min + 6 min	M + F	WT (shortfin)	12	→	→	
	Fluoxetine (1.2 mg/l)					→	↓	
	Fluoxetine (1 mg/l)					→	→	

Given are data pertaining to the drugs investigated (substances, doses, modes of administration), various important intervals (time allowed for drugs to work, habituation times and total test times), sex and strain. The following indices of anxiety-like behaviour are given: effects on (i) total time spent in the upper region (variously upper 1/3 or upper 1/2) – an increase here would usually be interpreted as an anxiolytic-like effect – and (ii) latency to first visit to the upper region; here a decrease is generally seen as an anxiolytic-like effect. ↑ indicates a significant increase, ↓ a significant decrease, → no significant differences and / that the parameter in question was not reported. The mode of administration was generally immersion and exceptions are indicated by either i.p. (intraperitoneal injection) or p.o. (peroral gavage). M + F indicates mixed-sex groups, ? that sex was not specified, M/F that males and females were tested separately and M or F that only males or females, respectively, were employed. A question mark under the ‘Strain’ heading indicates that no specification was given while WT (?) indicates that animals were described as wild-type with no further determination. In the paper by Sinyakova et al., strain was specified as ‘NTL’ with no further clarification.

**Table 2.** Reported effects of acute SRI administration in the scototaxis test using adult zebrafish

Table 2: Light-dark/scototaxis test		Time				Indices of anxiety-like behaviour		
Paper	Drugs (administration, doses)	Immersion (if applicable) +delay/habituation+test duration	Sex	Strain	N	Time in white portion	Latency to first entry	Notes
Benneh *et al*., 2017	Fluoxetine (0.03 mg/l)	20 min + 0? min + 5 min	?	WT (?)	?	→	/	Se note in table 1.
	Fluoxetine (0.1 mg/l)					→	/	
	Fluoxetine (0.3 mg/l)					→	/	
	Desipramine (10 mg/l)					→	/	
	Desipramine (30 mg/l)					↑	/	
	Desipramine (100 mg/l)					↑	/	
Fontana *et al*., 2022	Fluoxetine (100 μg/l)	30 min + 6 min + 6 min	M + F	WT (AB)	8	↑	/	
	Fluoxetine (100 (μg/l)	30 min + 0 min + 6 min				↑	/	No habituation, but 6 min of NTD test before LDB
	Fluoxetine (100 (μg/l)	30 min + 0 min + 6 min				↑	/	
Magno *et al*., 2015	Imipramine (0,5 mg/l)	10 min + 5 min + 15 min	M + F	WT (shortfin, longfin)	8 (16)	→	→	16 in the control group, 8 in the SSRI groups
	Imipramine (1 mg/l)					↓	↑	
	Imipramine (2 mg/l)					→	↑	
	Imipramine (4 mg/l)					→	→	
	Fluoxetine (1 mg/l)					→	→	
	Fluoxetine (2 mg/l)					→	↑	
	Fluoxetine (4 mg/l)					↓	↑	
	Fluoxetine (6 mg/ml)					→	→	
	Paroxetine (0,3 mg/l)					→	→	
	Paroxetine (3 mg/l)					→	↑	
Malyshev *et al*., 2021	Fluvoxamine (i.p. 5 mg/kg)	N/A + (10 + 10) min + 15 min	M + F	WT (shortfin)	18–30	→	/	Run immediately after novel-tank diving test.
Maximino *et al*., 2011	Fluoxetine (i.p. 5 mg/kg)	N/A + 30 min + 15 min	M + F	WT (shortfin)	10	→	/	
	Fluoxetine (i.p. 10 mg/kg)					→	/	
Maximino *et al*., 2013a	Fluoxetine (i.p. 2.5 mg/kg)	N/A + 10 min + 15 min	?	WT (longfin)	10	↓	↑	
	Fluoxetine (i.p. 5 mg/kg)					→	→	
	Fluoxetine (i.p. 10 mg/kg)					→	→	
Maximino *et al*., 2013b	Fluoxetine (i.p. 5 mg/kg)	N/A + (? min + 3 min) + 15 min	?	WT (longfin)	9–10	→	→	Se note in table 1.
	Fluoxetine (i.p. 5 mg/kg)			WT (leopard)		↑	→	
Maximino *et al*., 2014	Fluoxetine (i.p. 2.5 mg/kg)	30 min + 3 min + 15 min	?	WT (longfin)	10?	↓	→	Anxiolytic-like effect (normalised behaviour after schreckstoff)
Maximino *et al*., 2015	Fluoxetine (i.p. 1 mg/kg)	N/A + ? min + 15? min	M	WT (striped longfin)	9–10?	→	/	Se note in table 1.
Sabadin *et al*., 2022	Fluoxetine (100 (μg/l))	15 min + 0 min + 6 min	M + F	WT (?)	7	↑	/	Combined geo- and scototaxis test
Sackerman *et al*., 2010	Citalopram (100 mg/l)	3–4 min+(? min + 5 min) + 5 min	M + F	ZIRC AB, GloFish, WIC, PETCO (WT/shortfin?)	9	→	/	Black-white plus maze (after a 5 min novel-tank diving test), trend towards anxiolysis
	Desipramine (25 mg/l)				8	→	/	
Singer *et al*., 2016	Fluoxetine (10 mg/l)	3 min + 0 min + 4 min	M/F	WT (Scientific Hatcheries)	30	↑	/	Open field test

Given are data pertaining to the drugs investigated (substances, doses, modes of administration), various important intervals (time allowed for drugs to work, habituation times and total test times), sex and strain. The following indices of anxiety-like behaviour are given: i) total time spent in the white compartment - an increase here would usually be interpreted as an anxiolytic-like effect - and ii) latency to first entry to the white compartment; here a decrease is generally interpreted as an anxiolytic-like effect. ↑ indicates a significant increase, ↓ a significant decrease, → no significant differences and / that the parameter in question was not reported. In general, drugs were administered through immersion, though some studies employed intraperitoneal injection, as indicated by the abbreviation i.p. M+F indicates mixed-sex groups, ? that sex was not specified, M/F that males and females were tested separately and M or F that only males or females, respectively, were employed. A question mark under the ‘Strain’ heading indicates that no specification was given while WT (?) indicates that animals were described as wild-type with no further determination.

We found no previously published behavioural studies regarding fenfluramine and employing adult zebrafish. Therefore, a dose-finding study was conducted, with investigated concentrations (0.5 mg/l, 5 mg/l and 10 mg/l, i.e. 2.16 µM, 21.62 µM and 43.24 µM) being chosen on the basis of experiments in larval zebrafish (Zhang *et al*., 2015), rodents (Laferrere & Wurtman, 1989) and trout (Ruibal *et al*., 2002).

We did not observe any abnormal behaviour or any indications of negative effects on fish health during, or after, any of the experiments.

### Experimental outline

*Experiment I*: 27 animals (mixed sex, equal proportions of males and females) were randomised to immersion in either escitalopram in solution (50 mg/l) or fresh aquaria water for 10 min and then kept in separate aquaria according to the treatment group. Six weeks later, zebrafish were again treated according to the protocol above, with half of those in the control group now receiving escitalopram and vice versa. Animals were then subjected to a light-dark test.

*Experiment II*: 32 males and 12 females were randomised to immersion in either fenfluramine in solution (0.5 mg/l, 5 mg/l or 10 mg/l) or fresh aquaria water for 10 min. Animals were then subjected to a light-dark test.

*Experiment III*: 62 males and 37 females were randomised to immersion in either escitalopram in solution (25 mg/l or 50 mg/l) or fresh aquaria water, for 10 min. Animals were then subjected to a light-dark test.

*Experiment IV*: 60 males and 34 females were randomised to immersion in either escitalopram in solution (25 mg/l) or fresh aquaria water, for 10 min. Animals were then subjected to a light-dark test.

*Experiment V*: 52 males and 32 females were randomised to immersion in either fenfluramine in solution (5 mg/l) or fresh aquaria water, for 10 min. Animals were then subjected to a light-dark test.

Randomisation was done in a stratified manner by the first successfully netted animal from the first aquarium being assigned to treatment group A (actual treatment having been assigned by a third party and thus being unknown to the investigator), the second to treatment group B, the third to treatment group C (if three groups were included, otherwise to group A, etc. In the second aquarium, the order was reversed; in the third, the order was as in the first and so on. This was done to reduce the risk of baseline temperament factors being related to how easily an animal is netted (e.g. boldness) becoming unequally distributed among treatment groups, as well as ensuring that any factors relating to housing/aquaria position were equalised among treatment groups. Experimenters and caregivers were blinded to treatment status.

### Data analysis

Movies of each 15-min experimental session were manually analysed by an experimenter blind to the treatment condition of the animal. The following behavioural variables were assessed in each video: latency (s) to first entry to the white chamber, number of crossings between white and black chambers and total time (s) spent in the white chamber. These variables had been pre-specified. Apart from latency and total time, which had been commonly reported in earlier studies, we also included total entries to the light area, as reporting of this variable has been standard since the rodent light-dark box (i.e. the ancestor of the scototaxis test in zebrafish) was first developed (Costall *et al*., 1989). Due to a camera malfunction, the recordings of eight males (six escitalopram and two control) from Experiment III were not available for analysis.

### Statistical procedures

For analysis of behavioural parameters, two-way ANOVAs were employed, the exception being Experiment II where a one-way ANOVA with subsequent *t*-tests for relevant comparisons was used; as the variables were highly correlated, no correction for multiple comparisons was done. In Experiment I, treatment status at the first and the second sessions was set to be the predictive variable, while for Experiment III–IV, treatment and sex were employed. All analyses were done in SPSS for Mac, version 21 (IBM, Chicago, IL, USA).

## Results

### Systematic review

The search yielded 308 unique articles in total (304 in PubMed and a further 4 through Web of Science and Google Scholar). All articles found were written in English. All abstracts were then screened and reports clearly irrelevant to the research question were excluded, leaving 87 articles. The full texts of the remaining papers, having been identified as potentially relevant, were screened with a total of 28 papers being included. 24 papers had employed the novel-tank diving test (Benneh *et al*., 2017; Clément *et al*., 2020; Alves *et al*., 2023; Costa Iturriaga Vásquez *et al*., 2012; Maximino *et al*., 2015; Demin *et al*., 2017; dos Santos Sampaio *et al*., 2018; de Melo *et al*., 2019; Macrì *et al*., 2019; Giacomini *et al*., 2020; Lima-Maximino *et al*., 2020; Karakaya *et al*., 2021; Kulikova *et al*., 2021; Malyshev *et al*., 2021; Fontana *et al*., 2022; Maximino *et al*., 2015, 2013b, 2013a; Sackerman *et al*., 2010; Stewart *et al*., 2011; Sinyakova *et al*., 2018; Sabadin *et al*., 2013, 2022) and 12 the light-dark test (Magno *et al*., 2015; Maximino *et al*., 2015; Benneh *et al*., 2017; Malyshev *et al*., 2021; Fontana *et al*., 2014, 2022, 2013b, 2013a, 2011; Sackerman *et al*., 2010; Singer *et al*., 2016; Sabadin *et al*., 2022); some used both. The papers are summarised in Tables 1 and 2, Supplementary Table 1 and Figure 1. Regarding the SYRCLE analysis, we found most papers to have adequately controlled for baseline factors, as well as having addressed missing data in a satisfactory manner while not appearing to selectively report outcomes. Most studies employing the light-dark test also reported the outcome assessor to having been blind to treatment status. However, we found most papers to disclose insufficient details to permit the assessment of most items. We deemed the literature available for the novel-tank diving test to be comprehensive, and there is little need for any further experiments regarding the general influence of SSRIs in this paradigm. However, considerably less work had been done on anxiety-like behaviour in the light-dark test, despite some clear advantages compared to the novel-tank diving test, such as an apparent lack of risk of autonomic responses confounding any ethopharmacological effects (Finney *et al*., 2006; Sackerman *et al*., 2010). That no clear picture of the effects of SRI administration in the test emerged was then somewhat puzzling, although we did note that studies employing the test generally had used both low doses as well as very few SSRIs other than fluoxetine (with one exception (Sackerman *et al*., 2010)). All in all, there seemed to be enough outstanding questions to warrant further experiments.

**Figure 2.**
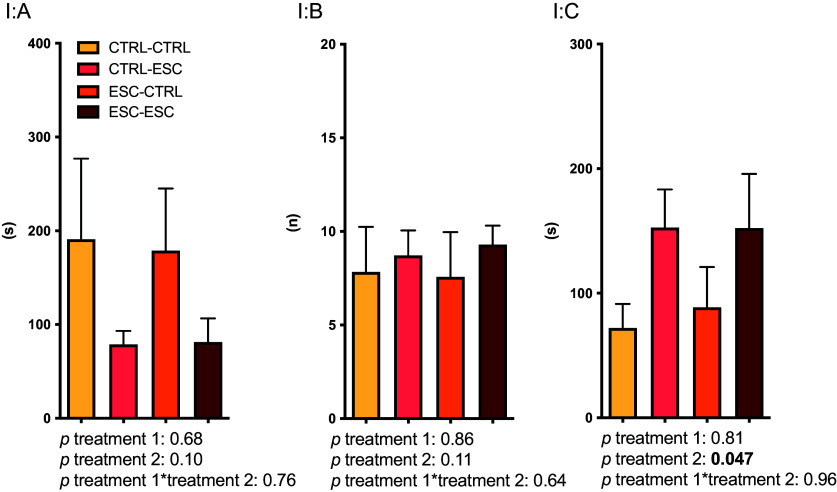
*Experiment I:* effects of escitalopram on latency to first entry into the white compartment (A), number of entries into the white compartment (B) and time spent in the white compartment (C) in the light-dark test. The test duration was 900 s. Given in the figure are the results of a two-way ANOVA with treatment status 6 weeks before and treatment status on the day of the light-dark session, respectively, as predictive variables. Error bars indicate SEM. Group sizes: *n* = 6 for (CTRL-CTRL), otherwise 7. ESC = escitalopram, CTRL = control.

*Experiment I:* No effects of previous single-dose exposure to escitalopram were seen on any of the indices investigated, and there were no treatment × treatment interactions. A significant, anxiolytic-like main effect for escitalopram treatment at session 2 on time spent in the light compartment was present (Figure 2).

**Figure 3.**
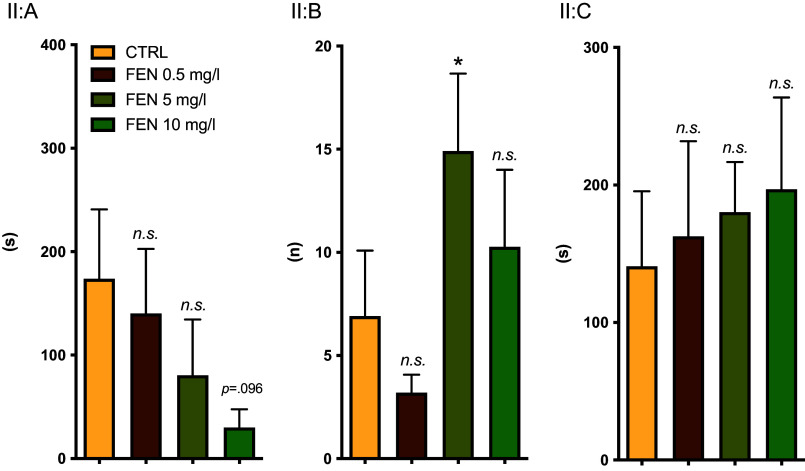
*Experiment II:* effects of fenfluramine (FEN) on latency to first entry into the white compartment (A), number of entries into the white compartment (B) and time spent in the white compartment (C) in the light-dark test. The test duration was 900 s. Indicated in the figure are *p*-values representing comparisons made to the control group. Error bars indicate SEM. Group sizes: *n* = 10 for FEN 0.5 mg/ml, otherwise 11. CTRL = control, *n.s.* = non-significant, * = *p < 0.05*.

*Experiment II:* A pattern suggesting a dose-dependent anxiolytic-like effect was observed albeit that a significant difference was only observed between the 5 mg group and the control animals in terms of the total number of entries into the light compartment (Figure 3). As we cannot discount the possibility that the reduction in the total number of entries at 10 mg/l as compared to 5 mg/l is related to effects on, for example, locomotion, the lower dose appears to be the better choice.

**Figure 4.**
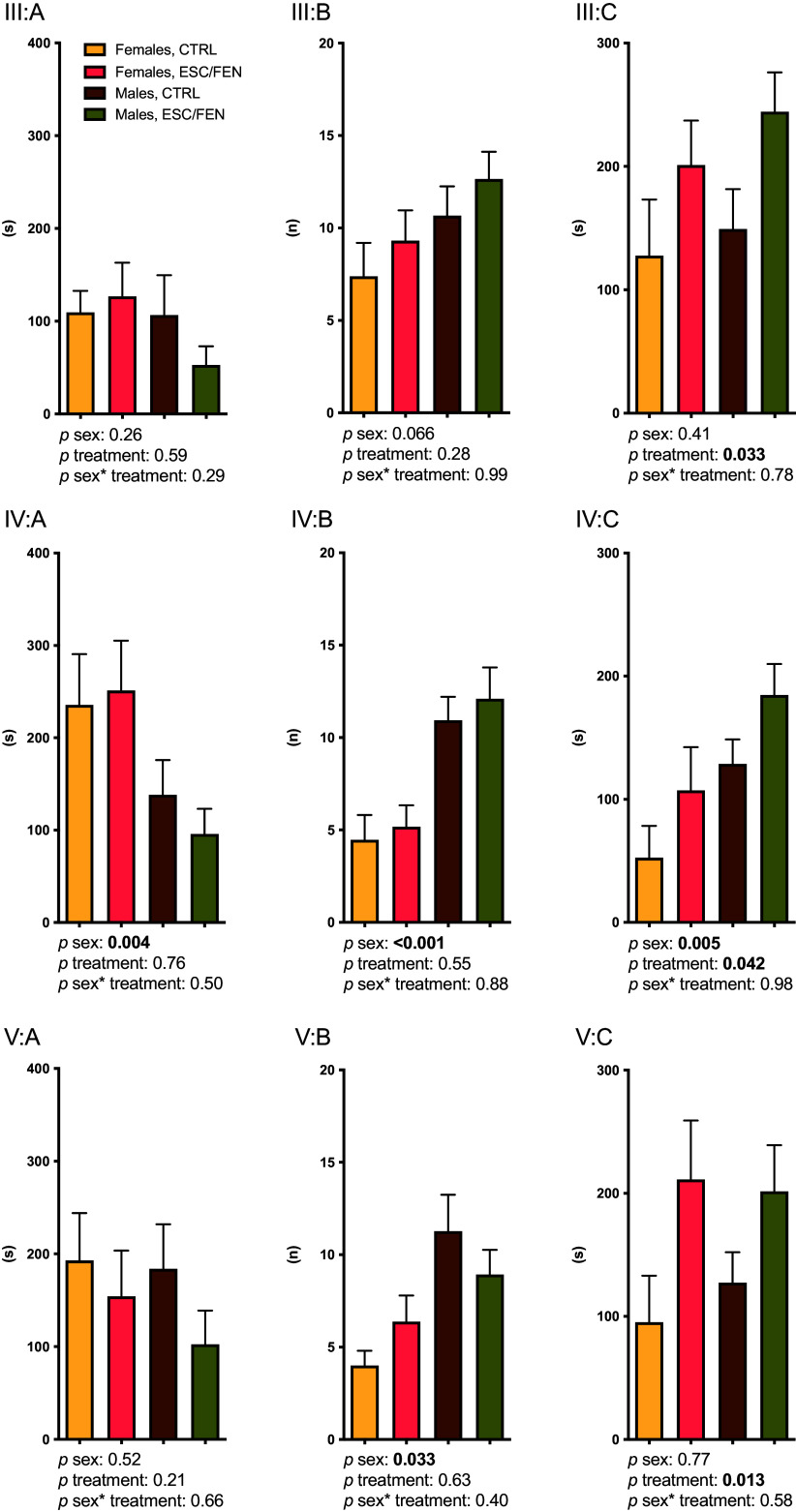
*Experiments III–V:* effects of escitalopram (ESC) (*Experiments III* and *IV*) and fenfluramine (FEN) (*Experiment V*) on latency to first entry into the white compartment (A), number of entries into the white compartment (B) and time spent in the white compartment (C) in the light-dark test. The test duration was 900 s. Given in the figure are the results of two-way ANOVAs with treatment and sex as predictive variables. Error bars indicate SEM. CTRL = control. Group sizes: *n* = 18 (males, control), 34 (males, ESC) 13 (females, control) and 26 (females, ESC) in *Experiment III*, 30 (males) and 17 (females) in *Experiment IV* and 26 (males) and 16 (females) in *Experiment V*.

*Experiment III:* No differences on any behavioural indices were observed between the two dose groups, and they were therefore collapsed into a single escitalopram group in all further analyses (Figure 4, panel 1). Significant main effects were seen for SSRI treatment on time spent in the white compartment (Figure 4, panel 1C), with animals receiving escitalopram spending more time in the light compartment. No effects on the other parameters recorded were observed, no sex effects were noted, and there were no significant interactions.

*Experiment IV:* As in Experiment III, a significant treatment effect on time spent in the white compartment was observed (Figure 4, panel 2C). A sex effect was present for all three recorded indices of anxiety-like behaviour, with males displaying less anxiety-like behaviour. There were no significant interactions (Figure 4, panel 2A-C).

*Experiment V:* Again, a treatment effect on time spent in the white compartment was present, with animals having received fenfluramine spending more time there (Figure 4, panel 3C). As in Experiment IV, a sex effect was observed, although only for number of entries to the white compartment. As in the earlier experiments, no significant interactions were observed (Figure 4, panel 3B).

## Discussion

Recalling the aims of the study as stated in the introduction, we would argue that our results (i) support the notion that acute administration of serotonin-releasing agents exerts an anxiolytic-like influence in the light-dark test in zebrafish and (ii) that this is not unique to reuptake inhibitors and (iii) that neither sex nor earlier exposure to the apparatus has any appreciable influence on treatment effects while (iv) earlier work strongly supports the existence of an anxiolytic-like effect of agents that increase synaptic serotonin in the novel-tank diving test while being more equivocal regarding the light-dark test.

Taken together, our findings would then indicate that the general effect of acute serotonin elevation, irrespective of whether this is achieved through blockage (escitalopram) or reversal (fenfluramine) of the serotonin transporter is anxiolytic, rather than anxiogenic, in zebrafish, as assayed by both the scototaxis and geotaxis tests. This is the opposite of the situation not only in humans (Grillon *et al*., 2007; Sinclair *et al*., 2009; Näslund *et al*., 2017), other mammals (Griebel *et al*., 1994) and birds (Warnick *et al*., 2009) but also in more distantly related metazoans such as crayfish (Fossat *et al*., 2014) (but note the anxiolytic-like effect in shore crabs [Hamilton *et al*., 2016]).

The neurobiology underlying a heightened capacity for anxiety/anxiety-like behaviour in humans and other mammals is only partly known, as are the long-term neurobiological effects of SRI administration beyond the immediate effects at the serotonergic synapse. It can nevertheless be noted that imaging studies indicate the 5-HT system in patients with anxiety disorders to be overactive (Frick *et al*., 2015) and that this can be attenuated by long-term SSRI treatment (Frick *et al*., 2016). Similarly, rodents displaying high anxiety-like behaviour have increased levels of the enzyme catalysing the rate-limiting step in serotonin synthesis, tryptophan hydroxylase 2 (TPH2), in the raphe nuclei, where serotonergic cell bodies reside, as well as higher serotonin levels in the amygdala as compared to less ‘anxious’ compatriots (Näslund *et al*., 2015). Finally, interventions that render rodents more ‘anxious’ also tend to raise raphe TPH2 levels (Chamas *et al*., 1999; Gardner *et al*., 2009; Sidor *et al*., 2010).

While differences, as compared to mammals, in ethopharmacological responses to SRIs conceivably could be related to the drugs having a partly different mode of action in teleosts, this does not seem to be the case (Winberg & Thörnqvist, 2016); acute SSRI treatment appears to increase serotonin turnover also in zebrafish. To our knowledge, no studies directly investigating the effects of fenfluramine on serotonin turnover in cyprinid brains, as measured by, for example, microdialysis, have been conducted. Nevertheless, it has been demonstrated to be a potent serotonin-releasing agent in mammals (Laferrere & Wurtman, 1989; Schwartz *et al*., 1989), as well as in rainbow trout (Ruibal *et al*., 2002).

When considering our results in relation to previously conducted studies identified in the systematic review (summarised in Tables 1 and 2), there is a clear agreement with results obtained from studies employing the novel-tank diving/geotaxis test (Table 1). Only one paper (Maximino *et al*., 2013b) reports an anxiogenic-like effect of acute SSRI administration and then only for one of several strains investigated. The addition of, for example, chronic unpredictable stress beforehand, or the presence of robotic predators or conspecifics does not seem to change this general picture (Clément *et al*., 2020; Costa de Melo *et al*., 2019; Karakaya *et al*., 2021).

It has been suggested that the observed anxiolytic-like effect of high (e.g. 100 mg/l of citalopram) doses of SSRIs in the novel-tank diving test partly reflects an effect on the swim bladder, unrelated to any effects related to affective behaviour (Finney *et al*., 2006; Sackerman *et al*., 2010), and while such an influence cannot be discounted without further investigation, similar behavioural responses are, as mentioned above, also seen at lower doses. It can be noted that in order to achieve brain tissue concentrations comparable to those in rodents and humans given standard doses of at least citalopram/escitalopram, comparatively high doses seem to be necessary; Sackerman and co-workers (Sackerman *et al*., 2010) estimate a citalopram concentration of 0.116 mg/kg in the zebrafish brain after a 3 min immersion of 100 mg/l citalopram in water. This can be compared to a study in mice reporting brain tissue escitalopram levels of 1121 nmol/l (0.363 mg/kg) 1 h after the administration of 5 mg/kg (Karlsson *et al*., 2013) or to post-mortem levels of 1.5–4 mg/kg in patients treated with citalopram and whose deaths were deemed to be unrelated to SSRI treatment (Nedahl *et al*., 2018). It may very well be the case that SSRI doses previously used in the light-dark test have been somewhat low, to the extent that comparisons can be made to rodent models and the clinical situation. In the case of fenfluramine, the maximum dose tested in Experiment I (10 mg/l) was comparable to that used when the drug had been tested as an antiseizure agent in larval models of Dravet syndrome (Zhang *et al*., 2015); we found no earlier studies investigating the effects of fenfluramine on behaviour in adult zebrafish and only one in fish at all (Weischer, 1966).

The light-dark/scototaxis test data is sparser and also more ambiguous (Table 2). It has been suggested that there exists a real discrepancy between the impact of acute SRI administration in the scototaxis and geotaxis tests and that this is related to a differential influence of serotonin on anxiety and fear/panic (Maximino *et al*., 2013a), in line with the Deakin–Graeff hypothesis; that is, that different subpopulations of serotonergic neurones exert differential effects on fear (or panic) and anxiety, respectively, with increased serotonergic tone inhibiting the former but promoting the latter (Paul *et al*., 2014).

In our opinion, it is however not immediately clear from an ethological and ethopharmacological point of view why one, but not the other, of the two tests should measure fear/panic rather than anxiety-like behaviour. It can be noted that while azapirones such as buspirone have some use in treating generalised anxiety disorder (Chessick *et al*., 2006) they are generally ineffective for panic disorder (Imai *et al*., 2014), a state of affairs that appears to be mirrored by rodent models (Graeff *et al*., 1996). In zebrafish they are effective in reducing anxiety-like behaviour in the scototaxis (Maximino *et al*., 2011, 2013a), as well as in the geotaxis test (Bencan *et al*., 2009; Maximino *et al*., 2013a), providing some support for both models to be regarded as primarily reflecting anxiety, rather than fear or panic, if one accepts the Deakin–Graeff hypothesis. Moreover, if the general influence of an acute increase of synaptic 5-HT levels in the scototaxis test would be anxiogenic, then a dose-response relationship could be expected for SRIs, but studies reporting such an effect of SRI administration find it to be transient and observed at only low or intermediate doses (Maximino *et al*., 2013a; Magno *et al*., 2015). Indeed, studies employing higher doses in the scototaxis test either report anxiolysis or trends theretoward (Maximino *et al*., 2013b; Sackerman *et al*., 2010).

Finally, the fact that it is *fluoxetine* in low doses that has been employed in all studies reporting an anxiogenic-like effect of acute SRI administration in the scototaxis test may be of some relevance, as low-dose fluoxetine has been reported to have minor effects on serotonergic neurotransmission in rodents while increasing levels of the GABAA-modulating neurosteroid allopregnanolone (Pinna *et al*., 2009). It is thus conceivable that an action on the neurosteroid system that is particular to this SSRI partly could explain these discrepancies. Also, while the clinical profiles of the SSRIs are generally very similar, the fact that fluoxetine is the least selective, in terms of monoamine reuptake inhibition, of the SSRIs (Sánchez & Hyttel, 1999), could possibly have a greater importance in zebrafish than in humans and rodents, given reported differences as compared to mammals in terms of affinity profiles of other reuptake inhibitors (Severinsen *et al*., 2008).

### Differences between the serotonin systems of mammals and teleosts

The effect of acute serotonin elevation on indices of anxiety-like behaviour is not the only instance where zebrafish differ from mammals in terms of central serotonergic functioning. Several other examples relating to anatomy, neuroendocrinology and behavioural pharmacology exist:

(i) Zebrafish, as well as other teleosts, have a population of serotonergic neurones in the hypothalamus, their relevance to behavioural pharmacology being unclear (Kaslin & Panula, 2001).

(ii) The teleost lineage underwent an early whole-genome duplication event early during its radiation, in addition to the two genome duplications shared by all jawed vertebrates (Volff, 2005). This means that zebrafish have an extra copy of also all genes coding for the entire molecular machinery of the serotonin system. Though not all extra copies remain functional, it is unknown if and then how this increase in the genomic complexity of the serotonin system (and the opportunity for specialisation it has afforded) affects ethopharmacological responses.

(iii) Cortisol responses to acute serotonin elevation are divergent, with acute SRI treatment inducing a decrease in blood cortisol in zebrafish (de Abreu *et al*., 2014) while an increase is typically seen in humans and other mammals (Hesketh *et al*., 2005; Ahrens *et al*., 2007).

(iv) Behavioural responses to a reduction in serotonergic tone differ. Serotonin depletion by way of *para*-chlorophenylalanine (*p*-CPA) robustly reduces anxiety-like behaviour while increasing aggression and dominance behaviour in mammals and birds (Miczek *et al*., 1975; Vergnes *et al*., 1986; Buchanan *et al*., 1994; Studer *et al*., 2015) while the effect in zebrafish seems to be the opposite, both in terms of anxiety-like behaviour (Maximino *et al*., 2013a; Müller *et al*., 2020) and aggression (Mezzomo *et al*., 2020). Some data exist on the effects of *p*-CPA treatment in other teleost species, and while cichlids (Adams *et al*., 1996) also respond with decreased aggressive behaviour, this is not the case with bluebanded gobies (Lorenzi *et al*., 2009) or cleaner wrasses (Paula *et al*., 2015).

(v) Responses to modulation of 5-HT2A/C receptors differ. In rodents, antagonism of these highly similar receptors is known to produce an anxiolytic-like response (Critchley & Handley, 1987; Griebel *et al*., 1997), while the opposite holds for agonism (Setem *et al*., 1999). 5-HT2A/C antagonism is also a commonality of several antidepressant and/or anxiolytic drugs such as agomelatine, mianserin and mirtazapine, and though ultimately not marketed due to an unfavourable safety profile, the 5-HT2C antagonist ritanserin was investigated as a treatment for generalised anxiety disorder during the late 80ies, with positive results in several randomised controlled trials (Ceulemans *et al*., 1985; Pangalila-Ratu Langi & Jansen, 1988). In zebrafish, acute administration of antagonists for these receptors increase (Nathan *et al*., 2015), while agonists attenuate (do Carmo Silva *et al*., 2021) conspecific alarm substance-elicited avoidant behaviour – but note a weak inhibition of post-exposure avoidant behaviour of antagonists (do Carmo Silva *et al*., 2021).

(vi) There are indications of differences in how baseline temperament relates to serotonergic functioning. A zebrafish strain displaying high anxiety-like behaviour has been reported to exhibit low levels of central 5-HT and low TPH2 activity (Maximino *et al*., 2013b) (but note the more complex picture presented by Tran and co-workers [Tran *et al*., 2016]), while as mentioned earlier, the association between humans and rodents seems to be in the opposite direction.

(vii) Unlike in rodents (Grigoryan *et al*., 2022), social isolation in zebrafish associates with *decreased* avoidance/anxiety-like behaviour in the scototaxis test (Shams *et al*., 2015; Varga *et al*., 2020) and is paralleled by increased serotonin release and turnover during the test (Varga *et al*., 2020) – but also with *lower* overall serotonin levels (Shams *et al*., 2015), although possibly only after short-term isolation (Shams *et al*., 2018).

Finally, the influence of the serotonin system on aggression deserves some elaboration. Notwithstanding the divergent responses to serotonin depletion, the effects of acute (and chronic) SSRI treatment in zebrafish seem to be similar to those in mammals and birds, that is, a reduction of aggressive behaviour (Sperry *et al*., 2003; Eriksson *et al*., 2008; Carrillo *et al*., 2009). However, available studies indicate that the effects of increased synaptic levels of serotonin on aggression may vary among teleosts as toadfish (McDonald *et al*., 2011) and matrinxã (Wolkers *et al*., 2017) respond to serotonin elevation with *increased* aggressive behaviour while Siamese fighting fish (Lynn *et al*., 2007) and blueheaded wrasses (Perreault *et al*., 2003), like zebrafish, respond with a decrease.

### The role of sex and habituation

Male zebrafish have been reported to display lower levels of anxiety-like behaviour but higher levels of locomotion as compared to females (Genario *et al*., 2020; dos Santos *et al*., 2021). We could observe the latter, but not the former, in Experiment III; however, a difference in terms of anxiety-like behaviour emerged in Experiment IV, with males being more ‘bold’. That this was observed in Experiment IV, but not V (the latter being conducted after thrice as long time as had elapsed between Experiments III and IV) could indicate that the sexes differ in terms of how they habituate to situations such as the scototaxis test – incidentally the only indication that habituation to the apparatus influenced our results in any way. In Experiment IV, females had markedly longer latencies to the first entry into the white compartment, while spending considerably less time there, as compared to the first session. This was not the case for males, and it was not the case for either sex in Experiment V. In no test did we observe an interaction between sex and treatment, indicating that sex has no substantive influence on the response to acute serotonin elevation. In our systematic review, we could identify only two studies where males and females had been directly compared in terms of behavioural SRI responses, with both employing the geotaxis test (or a variant thereof) and neither reporting a sex difference (Singer *et al*., 2016; Macrì *et al*., 2019). These findings are fully in line with what we observed.

Relatedly, we chose to house animals in sex-segregated aquaria. It has been reported that sex-segregated animals exhibit lower stress levels as indicated by cortisol levels (Reolon *et al*., 2018). In terms of behaviour, some minor effects on activity levels and anxiety-like behaviour have been observed in sex-segregated animals kept in unenriched, but not enriched, aquaria (Soares *et al*., 2020). As our aquaria were enriched, the net effect on behaviour is likely to have been small, if at all present, and overall stress levels should have been lower.

We found that most papers identified in the systematic review included insufficient information to assess the risk of bias (e.g. even though many mentioned randomisation, only two papers (Lima-Maximino *et al*., 2020; Alves *et al*., 2023) described the process in any detail) and to sometimes even omit basic information necessary for replication such as sex ratios and group sizes. In general, it would seem that the field would benefit from a more structured reporting of methodology. We recommend looking at, for example, the ARRIVE guidelines (du Sert *et al*., 2020) and also what is included in the SYRCLE assessment tool for systematic reviews (Hooijmans *et al*., 2014) not only when designing experiments but also during manuscript preparation in order to make sure that adequate information is provided while possible deviations necessitated by, for example, the condition modelled can be discussed in relation to such general guidelines.

### Strengths and limitations

The results of the behavioural experiments appear to be robust; group sizes were large, we controlled for sex and habituation effects and we also employed a potent serotonin-releasing agent not previously used in behavioural zebrafish research: fenfluramine. It has a partly different mode of action as compared to SSRIs and tricyclic antidepressants, and the finding that another class of serotonin-releasing drug produces the same result as agents that block the serotonin transporter strengthens the case that the behavioural effects we and others have observed are due to an acute elevation of synaptic serotonin.

Some limitations also deserve to be mentioned. Though individual differences in baseline behaviour are likely to influence behavioural responses to the agents used, we neither assayed baseline temperament differences nor tagged animals in order to track the performance of individual fish over the three experiments. Also, the choice to re-use animals may have had an influence on subsequent experiments. Nevertheless, the fact that we in Experiment I saw no indications of long-term effects of escitalopram treatment suggests that such fears are largely unfounded and that zebrafish, at least using drugs and setups similar to ours, can be re-used with appropriate drug wash-out times. Also, even if small such effects *would* be present, the impact then likely manifests itself as an increase in variance which, all else being equal, decreases the risk of false positives and increases the risk of false negatives. Thus, some caution might be advised when interpreting, for example, the absence of effect in Experiments IV and V, but on the other hand, the main effect observed, that is, a general anxiolytic-like effect as indicated by an increase of total time spent in the white compartment in all three experiments (as well as in Experiment I), is then likely very robust.

## Conclusion

To summarise, we have in a series of experiments observed a consistent anxiolytic-like effect of serotonin-releasing agents in zebrafish in the light-dark test. After reviewing the available literature, we found that there is robust evidence for an anxiolytic-like effect in the other major zebrafish model of anxiety, the novel-tank diving test. We also found that a number of studies indicate other notable differences in terms of how the serotonin system influences behaviour in zebrafish as compared to mammals. This would appear to be most pronounced in behavioural responses to short-term serotonin elevation or depletion, while responses to 5-HT1A agonists (Maximino *et al*., 2013a; Magno *et al*., 2015) and long-term SRI treatment (Egan *et al*., 2009; Magno *et al*., 2015) are more similar, but differences also seem to be present in terms of neuroendocrinological responses to manipulation of the serotonin system and, to some extent, the relationship between serotonin and temperament. Whether this extends to related taxa is unclear. It is hard to say how representative zebrafish are in terms of behavioural responses to, for example, acute serotonin elevation in cyprinids, or teleosts, in general – as noted, the effects on aggression seem to vary in the handful of species investigated. In the case of anxiety-like behaviour we are aware of a single study, in piauçu (Barbosa *et al*., 2012), where acute SSRI administration has been investigated in fish other than zebrafish; it was found to decrease antipredator behaviour, that is, a finding in line with observations in zebrafish.

Though our investigation has focused on short-term interventions, these (and other) indications of differential functioning in terms of the 5-HT system suggest that caution is prudent also when interpreting the effects of long-term treatment, as the mechanisms underlying the positive therapeutic effects of SRI administration in humans are poorly known. It cannot be taken for granted that a similarity of zebrafish and mammalian behavioural responses to chronic SRI administration also reflects a high degree of similarity in terms of underlying neurobiological mechanisms. In our opinion, the discrepancies we discuss in this paper warrant further investigation as they potentially call into question the translational value of research into affective disorders that employ zebrafish as a model organism. Of lesser practical, but not scientific, value is also further investigation of variation between species in terms of serotonergic functioning and how such variation may relate to ecological roles and evolutionary relationships.

## Supporting information

Näslund et al. supplementary material Näslund et al. supplementary material

## Supplementary material

The supplementary material for this article can be found at https://doi.org/10.1017/neu.2024.44.
